# The forgotten art of cold therapeutic properties in cancer: A comprehensive historical guide

**DOI:** 10.1016/j.isci.2023.107010

**Published:** 2023-05-29

**Authors:** Tatiana P. Grazioso, Nabil Djouder

**Affiliations:** 1 Molecular Oncology Programme, Growth Factors, Nutrients and Cancer Group, Centro Nacional de Investigaciones Oncológicas, CNIO, ES-28029 Madrid, Spain; 2 Gynecological, Genitourinary and Skin Cancer Unit HM, Clara Campal Comprehensive Cancer Center, CIOCC, Department of Basic Medical Sciences, Hospital Universitario HM Sanchinarro, ES-28050 Madrid, Spain; 3 Institute of Applied Molecular Medicine, IMMA, Facultad de Medicina, Universidad San Pablo CEU, ES-28668 Madrid, Spain

**Keywords:** Medicine, Systems biology, Cancer

## Abstract

Cold therapy has been used for centuries, from Julius Caesar to Mohandas Gandhi, as a potent therapeutic approach. However, it has been largely forgotten in modern medicine. This review explores the history of cold therapy and its potential application as a therapeutic strategy against various diseases, including cancer. We examine the different techniques of cold exposure and the use of other therapeutical approaches, such as **cryoablation, cryotherapy, cryoimmunotherapy**, **cryothalectomy,** and delivery of **cryogen agents**. While clinical trials using cold therapy for cancer treatment are still limited, recent research shows promising results in experimental animal cancer models. This area of research is becoming increasingly significant and warrants further investigation.

## Therapeutic application of cold through history

### “Knowledge gained from practical experience”

The Edwin Smith Papyrus, written around 2000 to 2500 BC during the Pyramid Age, is the oldest known book on medical science and surgery (medical and surgical treatise), and describes the “f*ascinating revelation of the human mind struggling with the first stages of science-building*.”^1^ The Edwin Smith Papyrus presents detailed anatomical, physiological, pathological, and surgical descriptions, recorded in 48 cases, each consisting of a **medical ailment** with a detailed description of the examination process of the patient, as well as the diagnosis, cause, and prognosis (treatable, uncertain, and non-treatable). Furthermore, based on the case prognosis, a suggested treatment, including surgery, medical remedies, procedures, or instructions to “*let nature do the work*,” is described in detail.^1^^,^^2^ Interestingly, and contrary to other medical papyri of the time, including the Kahun Papyrus, Ebers Papyrus, Hearst Papyrus, and Ramesseum Papyri, the medical procedures and treatments described in the Edwin Smith Papyrus were rational and surgical. Indeed, they almost entirely excluded the use of magic and religious healing practices, and were solely based on observation leading to medical conclusions, thus representing the first known “*evidence of an inductive process in the history of the human mind*.”^1^

**Figure 1:**
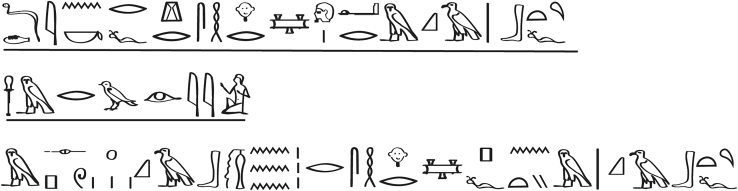
Diagnosis of case 46 extracted from the Edwin Smith Papyrus This translates as follows: *“One having an abscess with prominent head in his breast. An ailment which I will treat with cold applications to that abscess which is in his breast*.” Adapted from,^1^ extracted from the Edwin Smith Papyrus exposed at the New York Academy of Medicine, NY, USA.

Interestingly, among the 48 cases described, case 41, entitled “An infected or possibly necrotic wound in the breast,” and case 46, entitled “An abscess with a prominent head on the breast,” describe the earliest evidence of the use of cold to treat human disease.^1^ At a deeper level, the medical treatment of case 41 is elaborated; the first step of treating a man who presents with fever due to a diseased inflamed wound in his breast involves the local application of cold “*for drawing out the inflammation from the mouth of the wound*.” Furthermore, the examination and diagnosis of case 46 describes a soft swollen abscess with a prominent head in the patient’s breast, which secretes an oily fluid or pus, but with no redness; the treatment consisted of repeated cooling applications (Figure 1). However, in cases where cold applications were ineffective, wound healing with acacia, sycamore leaves, or other herbal remedies were applied to the wound.^1^

### “Knowledge gained from observation”

Following the Egyptians, 1000 years later, in 400 BC, the ancient Greeks believed that the body areas with an excess of heat were those presenting the disease, and considered that this excess of heat was the cause of the ailment. Consequently, they believed that inducing **hypothermia** could be a useful therapeutical approach to dispel the heat. Hippocrates was the first to induce hypothermia in patients, noticing the analgesic benefits of cold, and documented the use of cold for medicinal purposes, stating that “*water can cure everything*” in his “On Airs, Waters, and Places” treatise.^3^^,^^4^^,^^5^^,^^6^ Strengthening his beliefs, Hippocrates observed that infants who were exposed in the open survived longer in winter than in summer,^7^ attributing these health benefits to cold. He also explained that cold reduced swelling and dispelled pain, and suggested that cold should be applied around hemorrhages by packing wounded patients in snow and ice.^2^^,^^3^^,^^8^^,^^9^

Later, the Hippocratic School of Medicine returned to this technique by inducing whole body cooling techniques to treat systemic diseases.^2^ Similar to this practice, the Homer’s Iliad, filled with human war health crises, mentions the use of cold to treat war wounds. One such example is the description of how Eurypylus, a Greek warrior wounded by an arrow, was treated by Patroclus, who cut the arrow, washed the wound with cold water, applied some herbs and bandaged it, allowing the wound to dry, and the bleeding to stop.^10^

Later, the ancient Romans recommended induced cooling for battle-inflicted trauma or cerebral disturbances, and used both local and systemic cooling methods to treat patients presenting with fever and infections.^7^^,^^11^ Julius Caesar used ice-cold treatments to relieve ailments, and **cold-water immersion** was commonly used by the Roman physician Claudius Galen to treat tertian fever.^4^^,^^10^ Later on, throughout the Middle Ages, the same principle of cold-water immersion was frequently used to alleviate fever, and the physician Girolamo Mercuriale recommended cold chilled spring water baths to “*wash away the pain*.”^2^ Similar practices were reported in the new world, where, in the 1500s, Amerigo Vespucci reported how natives on the northern coast of Honduras used cold baths to reduce fever, stating, *“… for many times I saw that with a man sick of the fever when it heightened upon him, they bathed him from head to foot with a large quantity of cold water …”*.^12^

### “Knowledge gained from duality”

Many ancient Mesoamerican civilizations believed in duality, where everything had two opposing sides (good/bad, light/darkness, hot/cold), and harmony from chaos was only reached upon finding the balance between this duality. These beliefs were shared in their medical practices, not only to understand the illness and its cause but also to understand how to treat it.^13^ Considering this duality, they believed that to be healthy, the body requires a balanced equilibrium of hot and cold, and that an excess of either one would favor disease; thus, remedies of the opposite properties would be necessary to reestablish this balance^14^ (Table 1). The Maya civilization, during the search for duality, classified several illnesses, as well as the medicines, plants, and foods used to treat these ailments, into “cold” and “hot.” In this regard, “hot” illnesses, such as fever, diarrhea, and vomiting, required a cold treatment, and thus cold and cold plants and foods were used to treat the disease. Conversely, “cold” illnesses, such as constipation and paralysis, were treated with heat and spicy foods, such as pepper and garlic. Importantly, to maintain the equilibrium between this duality (termed “fresco”), they used neutral plants and foods, which were believed to be nutritious and vitamin-rich.^15^^,^^16^

**Table 1:** Hot-cold classification of illnesses and medicinal plants (Adapted from Alvarez-Quinoz et al., 2016)

TYPE	Disease/Symptoms	Type	Plants used to treat
HOT	Burns, wounds, skin dryness, and snake bites	**COLD**	*Theobroma cacao L.* Cocoa
HOT	**Cancer**, burns, and wounds	**COLD**	*Blechum pyramidatum* Cancerillo
HOT	Inflammation and stomach pain (empacho)	**COLD**	*Manilkara zapota* Chicozapote
HOT	Inflammation	**COLD**	*Pelargonium Citrosum* Citronella
HOT	Burns, diarrhea, and urinary problems	**COLD**	*Cocos nucifera L.* Coconut
HOT	Fever, diarrhea, stomach pain, and dysentery	**COLD**	*Annona reticulata L.* Soursop
HOT	Fever, diarrhea, parasites, and inflammation	**COLD**	*Mentha x piperita L.* Peppermint
HOT	**Cancer**, diabetes, and inflammation	**COLD**	*Kalanchoe gastonisbonnieri* Mala madre
HOT	Diarrhea, pimples, and blackheads	**COLD**	*Annona reticulata L.* Anona
HOT	Parasite, gastritis, and asthma	**COLD**	*Carica papaya L.* Papaya
HOT	**Cancer**	**COLD**	*Dioscorea composita* Barbasco- Ñame morado
COLD	Cold, rheumatism, and evil eye (mal de ojo)	HOT	*Citrus limon L.* Lemon
COLD	Stomach pain (empacho) and sterility	HOT	*Matricaria chamomilla L.* Chamomile
COLD	Heart problems, low blood pressure, and cough	HOT	*Allium sativum L.*Garlic
COLD	Cough	HOT	*Cinnamomum zeylanicum* Cinnamon
	Burns, wounds, inflammation, hemorrhoids, colitis, gastritis, and ovary pain	HOT and COLD	*Sábila Aloe vera L.Aloe vera*

Other Mesoamerican civilizations, such as the Aztecs and the Zapotecs, followed similar medical practices based on duality and balance beliefs,^16^^,^^17^ where “hot” diseases were commonly associated with fire and thus treated with cold, predominantly involving water.^13^ For instance, Nahuas from la Sierra de Texcoco in Mexico believed that diarrhea was a “hot” disease, which unbalanced the thermal balance of the body resulting in fever, dehydration, and sweating; thus, teas made from “cold” and “fresh” plants such as mint and spearmint were given as cold treatments to cure the disease.^18^ Importantly, this traditional medicine was inherited from generation to generation, and hot and cold plants are still widely used today, representing a promising approach for identifying new drugs and antitumoral agents.

### “Knowledge gained from observation and experimentation”

This practice continued in the 1700s when during the fever epidemic in Bratislava, cold water baths were used to fight and destroy the element of fire causing the fever.^12^ However, it was not until 1766 when Dr. John Hunter, known as the father of modern surgery, began complete and methodical experiments on hypothermia and cold exposure, believing that he could only understand life upon death, and considering that the closest state to death was hypothermia. His studies described how living beings generate heat upon cold, body temperature regulation, and the idea of offering eternal life by inducing hypothermia. Importantly, his studies on the effect of cold on animal hearts and his resurrection methods form the basis of modern cardiopulmonary resuscitation.^2^

In 1778, the medical student James Currie addressed the Royal Medical Society of Edinburgh, with “What are the effects of cold on the living body?” highlighting his interest in this matter, which followed him throughout his career.^12^ However, it was not until 1792, after observing 11 crew members who survived a shipwreck after being immersed in cold seawater, while those exposed to wind and rain perished, that he became fully interested in the effect of cold. Following this observation, he induced hypothermia in humans by cold-water immersion^2^^,^^19^ to study the impact of cold water on human physiology, with the aim to understand its effect on body temperature, pulse, respiration, and other parameters. His studies were of great importance as he described that cold had both stimulant and sedative effects in humans, and showed how the heart rate is reduced after exposing human volunteers to prolonged cold-water baths. Importantly, his studies formed the basis of peripheral vasodilation upon **thermoregulation**, and are still used today to decrease the heart rate of patients before surgery.^4^^,^^12^^,^^19^

Furthermore, after applying different cooling methods to patients presenting multiple clinical disorders, James Currie documented for the first time that the main function of **perspiration** is to regulate body temperature in health and disease.^4^^,^^12^^,^^20^ Similar to ancient practices, James Currie and Dr. William Wright advocated the scientific use of cold water to decrease febrile stages,^12^ a practice that is still used today.

The understanding of the benefits of cold exposure deepened throughout the 19^th^ century, where great scientists, such as Thomas Bell, John Hughes Bennet, James Arnott, Thomas Weedon, J.W. Bright, and Silas Weir Mitchell, displayed interest in the effect of cold in disease and reported favorable responses in multiple clinical conditions (pain, swelling, tumor formation, and inflammation) after local cold application.^21^ James Arnott described the multiple benefits of local cold application to treat headaches and neuralgia, and together with John Bennet, who believed and pioneered that cold application could retard the advancement of cancerous growth, described the therapeutic benefits of applying cold in external cancers.^22^^,^^23^ Hypothermia was also used by William Osler, who decreased the mortality rate of patients with typhoid from 24.2% to 7.1% at the Johns Hopkins Hospital by using body cooling regimens.^20^

Later, in 1803, the Russians used cold for multiple medical reasons, including covering patients in snow, with the aim to return spontaneous circulation.^24^ During the war of 1812, Napoleon’s surgeon, General Barón Larrey, observed that injured soldiers that remained close to the fire died faster than those in a hypothermic state.^10^ During Napoleon’s Russian campaign, he used ice and snow to preserve injured limbs, and as a form of anesthesia for performing painless amputations and other surgical procedures upon soldiers in the field.^20^^,^^24^

The curative properties of cold were also used extensively to treat multiple mental illnesses in the 19^th^ and 20^th^ centuries.^2^^,^^25^ As previously mentioned, Hippocrates believed in the power of cold and water, and presented the Hippocratic “humors theory,” which describes that each state of mind corresponds to an element (fire, air, water, and earth). Considering this theory, mental illness was believed to be a disease caused by an excess of heat in the brain. Following this idea, the physician Philippe Pinel, in 1971, reported several cases showing favorable effects of hypothermia on mental illness after observing that a young man with mania who escaped from an asylum and spent the night in a snowy forest, entering into a hypothermic state, was cured of his mania upon recovering. Later, John Talbott studied the effect of cold water in patients with schizophrenia but failed to obtain conclusive results.^2^^,^^20^ Indeed, no conclusive results were obtained regarding the use of cold to treat mental disorders. Although the effect of hypothermia on mental illness remains unclear, cold exposure is now known to activate the sympathetic nervous system, increasing noradrenaline release in the brain, and beta-endorphins and noradrenaline levels in the blood, which could result in an antidepressive effect.^26^ More recently, frequent ice-cold water swimming and immersion have been shown to replicate these observations.^27^ Regardless of the advanced knowledge gained during this period, therapeutic hypothermia never gained the correspondent recognition regardless of its advances and promising results and was somehow forgotten over time.

## Therapeutic application of cold in cancer treatment

### “Knowledge gained from experimentation”

A more extreme use of cold in the clinic involves using freezing temperatures to destroy tissue sections. In 1845, Dr. James Arnott believed that certain diseases that had been shown to resist conventional methods, such as cancer, were “*not necessarily fatal or incurable by the powers of Nature or Art*.”^22^ By these means, he employed congelation (−18°C to −24°C) (later known as **cryotherapy**) by applying iced salt solutions to freeze advanced breast and uterine cervix cancers; strikingly, he reported tumor regression and pain amelioration, stating that *“congelation arresting the accompanying inflammation, and destroying the vitality of the cancer cell, is not only calculated to prolong life for a great period but may also, not improbably, in the early stages of the disease, exert a curative action*.*”*^23^ Moreover, he observed that pain relief upon local congelation was more efficient than the use of opium, and presented fewer side effects, which led him to continue his studies on the anesthetic properties of cold and refrigeration anesthesia.^22^^,^^23^ In the 19^th^ century, upon liquefying gases such as oxygen (−182.9°C), nitrogen (−196°C), and hydrogen, cryogen agents arose for therapeutic purposes, mainly to treat skin diseases such as small skin cancers, pigmented nevi, lupus, and epitheliomas.^23^

### “Knowledge gained from methodical experimentation in modern times”

The first methodological studies of hypothermia in cancer are attributed to Dr. Temple Sedgwick Fay, who, in 1919, became interested in the effects of low temperature on cellular growth and cancer after observing that metastasis was more frequent in high-temperature body areas. His initial research showed that *in vitro,* chicken embryonic growth and cell differentiation inhibition occurred at 0°C. Upon this observation, he hypothesized that if normal undifferentiated cells required a normal temperature to complete division, the same must apply to undifferentiated cancer cells. To understand this principle, he investigated the effect of applying different temperatures to both normal and cancer cells. In accordance with his hypothesis, he observed that temperature affected undifferentiated malignant cell differentiation, such that 32°C exposure was sufficient to almost completely cease malignant cell differentiation. Importantly, his studies showed that healthy differentiated cells had greater cold tolerance than malignant undifferentiated cells.^2^^,^^28^^,^^29^

Based on these observations, in 1938, Dr. Fay began human trials on systemic and local hypothermia to treat malignancies and head injuries. First, he treated a young woman with metastatic ulcerated breast cancer and debilitating pain by applying local refrigeration for several weeks to the ulcerated tumor. Repeated biopsies over time demonstrated tumor regression, infection clearance, and wound healing upon cold, and further biopsies showed the complete disappearance of the neoplastic process after 5 months.^28^^,^^29^ Moreover, patients with terminal cancer previously exposed to surgical and radiation treatments, with survival odds of 1:8 and a life expectancy of only a few weeks, were exposed by Dr. Fray to whole-body hypothermia under tribromoethanol anesthesia. To decrease core body temperature (CBT), he allowed nature to supply cold, reaching air room temperatures of 14°C together with whole body exposure to chopped ice; using this method, Dr. Fray’s patients reached CBTs of 32°C. Surprisingly, he recorded 95.7% of pain reduction and a drop in mortality to 10%; patients not only survived longer but also had a better quality of life than predicted.^2^^,^^29^

However, his methods were considered radical and dangerous, favoring weak respiration and pulse. Thus, to decrease the problematic methodology of CBT reduction, Dr. Fray developed special cooling blankets that pumped cold water solutions through rubber tubing using discarded CO_2_ gas capsules and beer-cooler machines, which he used to wrap patients to decrease their CBT. He also developed and implanted metal capsules or “**ice bombs**” with connecting tubes to apply localized cryotherapy, allowing circulating refrigerants to treat localized brain lesions after intracranial tumor resection or after trephine opening of the skull. Several of the 123 patients with cancer who were implanted with Dr. Fray’s ice bombs showed degenerative changes (tumor regression) in the affected area and a lack of inflammation around the capsule, supporting the antitumoral effects of cold.^2^^,^^21^ Biopsies obtained from these patients revealed that the brain tissue tolerated subnormal temperatures, even to levels of freezing, without affecting its cellular structure (beyond 2 mm of the capsule); however, the adjacent tumor areas presented degenerative changes and a lack of inflammation around the capsule.^21^

He continued his studies using localized refrigeration delivered by different devices to routinely apply cold to patients presenting with cerebritis, brain abscess, and osteomyelitis of the skull. Often, after open surgeries for brain abscesses and cerebritis, he directly irrigated refrigerated saline and boric acid or Dakin’s solution into the infected area, noting satisfactory responses in both infectious and neoplastic diseases. Furthermore, for severe head injuries, he developed a deliberated hypothermia treatment, which involved reaching 23.88°C for 3 days; impressively, the patient fully recovered after the treatment.^2^^,^^20^^,^^21^ At a deeper level, he described that after traumatic brain injuries, hypothermia favored a better utilization of oxygen by the brain tissue while decreasing intracranial pressure, thus favoring a positive outcome.^2^

Dr. Fay was the first to show that hypothermia reduced inflammation, swelling, and edema; cleared infections (without the use of antibiotics); and retarded human malignant cellular metabolism and growth, favoring tumor regression. Moreover, when applied locally, hypothermia led to tumor regression in cutaneous cancer metastasis, although the mechanisms remain unclear.^2^^,^^21^ Fay also conducted methodological experiments, which provided the foundations of hypothermia as a therapeutic approach for cancer and neuroprotection.^2^ Similar to Dr. Fray, to treat cerebral neoplasms, Klikov developed a head blanket in which ice-cold water circulated, which reduced deep brain temperatures from −18°C to −20°C in small laboratory animals.^30^

**Cryoablation** arose in later years, and many techniques were developed to precisely freeze and ablate tissues. Apparatuses were cooled by pressurized carbon dioxide, freezing mixtures were circulated in cannulas (mainly solid carbon dioxide in acetone [–74°C]), and liquid cryo-agents such as liquid nitrogen showed promising clinical applications to remove frozen tumoral-brain tissue.^23^^,^^30^ However, it was not until 1961 when neurosurgeon Dr. Irving Cooper developed the first cryoprobe system to perform cryothalectomies, inspired by a carbon dioxide wine bottle opener, which not only cooled his hand but also looked like a brain cannula. Together with the engineer Robert Johnson and the cryobiologist Arthur Rinfret, Dr. Cooper designed the first closed cryosurgical probe, a vacuumed-insulated probe that allowed controlled freezing of body tissues by liquid nitrogen (−196°C).^31^^,^^32^ His design aimed to produce cryogenic lesions in the basal ganglia and thalamus in the brain to treat Parkinson (with a success rate of 90% in good-risk patients and 60% in poor-risk patients) and other neurological diseases.^20^^,^^31^^,^^32^ Intrigued by the advantages of cold presenting anesthetic, coagulant, and destructive properties, he recognized the usefulness of cryosurgery for treating tumors and studied the properties of congelation and necrosis in intracranial tumors and other body parts.^23^^,^^31^ He observed that in reachable brain tumors, intracerebral cryosurgery favored tumor freezing and tumoral shrinkage, facilitating its dissection. He also described the necrotic properties of cryothalectomies upon inoperable, deep intracerebral gliomas and other brain tumors. Moreover, he showed that 5 min at −100°C to −160°C favored rectal tumoral shrinkage and necrosis, stopped rectal mucous discharge and bleeding, and favored an immediate relief of local symptoms.^31^ Even though his work and procedures were scientifically criticized, he became a pioneer in modern cryogenic surgery.

Today, cryosurgery can be monitored by modern imaging for precise and control zone ablation and is widely used in the clinics, showing beneficial responses in skin, liver, breast, renal, and prostate cancer alone or in combination with chemotherapy, immunotherapy (such as immune checkpoint inhibitors), radiotherapy, or embolization.^33^^,^^34^ Furthermore, recent studies performed in mouse models have shown how cryoablation not only destroys neoplastic tissues but can also generate a systemic tumor-specific immune response, enhancing the ability of the immune system to recognize and attack cancer cells both locally and systemically, eradicating micro-metastases.^35^^,^^36^

**Figure 2:**
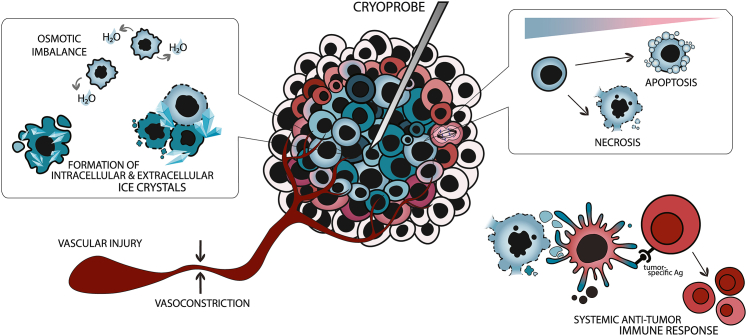
Cryoablation: mechanism of action and tumor-specific immune response

Mechanistically, cryoablation uses liquid cryogens or an ablation probe needle (cryoprobe), to apply extremely cold temperatures obtained from circulating cooled fluids to freeze and ablate tissue sites precisely and locally. Briefly, this mechanism results in coagulative necrosis, osmotic imbalance favoring cell dehydration and concentrating toxic electrolytes, and the formation of ice crystals (−20°C) in the extracellular space, causing cell membrane damage and cell rupture. Importantly, necrotic cells induce cellular stress signals and release tumor-specific antigens, type 1 cytokine, and damage-associated molecular patterns, thus stimulating systemic antitumor immune response (most likely by enhancing tumoral-specific T cell response, as observed in mouse models).^31^^,^^33^^,^^35^^,^^36^ Interestingly, this immune response enhancement upon localized breast cancer cryotherapy has been associated with the **abscopal effect** that can trigger a systemic immune response that leads to the regression of the distant, untreated tumors.^34^^,^^35^ In other words, the immune system is activated by the localized treatment and starts attacking cancer cells throughout the body. Moreover, freezing temperatures disrupt cells and mitochondrial metabolic functions, triggering Bax proteins and apoptotic pathways. Finally, cold favors vascular stasis, decreasing the tumoral blood supply, favoring tumoral hypoxia and cell damage^31^^,^^33^^,^^35^^,^^36^ (Figure 2).

Cryoablation is thus not only effective in inducing cold-induced cytotoxic effects with minimal side effects, but as mentioned, due to the abscopal effect, it can also enhance the sensitivity to immune checkpoint inhibitors.^35^^,^^37^ Immune checkpoint inhibitors block checkpoint proteins such as PD-1, PD-L1, and CTLA-4 that suppress T cell effector mechanisms and prevent immune cells from attacking cancer cells.^38^ For instance, cryoablation enhances anti-CTLA4 antibody therapy where 80% of mice are tumor free, and currently phase II trials combining cryoablation with ipilimumab and nivolumab (anti-CTLA4 and anti-PD1) for patients with triple-negative breast cancer are ongoing, supporting the synergistic antitumor effects of cryoablation and immunotherapy.^37^^,^^39^ Based on these observations, cryoimmunotherapy has gained more attention, and new studies using nanocarriers for cryoablation therapy are arising,^33^ representing an exciting approach in the field of cancer treatment.

Lastly, some of the most promising responses to cryoablation have been observed in clinical trials for breast cancer and focal metastatic sites. In 2004, ultrasound-guided cryoablation performed in 27 patients had a 100% success rate in primary invasive breast cancers of ≤1 cm and a 63% success in patients with >1 cm.^40^ Similarly, a 75.9% success rate was observed in phase II ACOSOG z1072 trial, where 86 patients with unifocal invasive ductal carcinoma (≤2 cm) underwent cryoablation.^41^

### “Misused knowledge”

In 1942, Dr. Frayś manuscripts were confiscated by the Nazis in Belgium, who used Dr. Frayś advances in hypothermia research to implement the Nazi immersion-hypothermia project performed at the Dachau concentration camp, which consisted of 30 projects involving the use of hypothermia in humans.^42^ Due to the horrors of medical experimentation during this period, hypothermia research interest took a step back. Many years passed until a resurgence in therapeutic hypothermia due to its beneficial neuroprotective role described in several clinical trials.^2^^,^^43^ Today, modern medical practices use cold for ablating tumors with extreme cold exposure (cryoablation), for hypothermic circulatory arrest to ease cardiac arrest, for neuroprotection, and to maintain normothermia.

### “Knowledge obtained from molecular and biological experimentation”

As described, cold exposure aims to achieve a systemic or local reduction of CBT, reaching, to some degree, a hypothermic state. However, cold exposure may not always reach a hypothermic stage, favoring instead whole-body or local cooling. Nevertheless, multiple data support a wide range of therapeutical properties involving the use of cold, and even though the mechanisms are yet unclear, cold exposure and lowering CBT have been proven to improve lifespan, aid hepatic enteropathies, neuropathies, brain and spinal cord trauma, cardiac arrest, neonatal hypoxic enteropathies, and ischemic injuries.^24^^,^^44^^,^^45^ Although the therapeutic role of cold in cancer is auspicious, it remains limited and unclear, making it an exciting subject for future investigation.

## Effect of cold on immune responses, cell proliferation, metabolism, and metastasis

One of the first attributes of the therapeutic role of cold is its ability to decrease inflammation and inhibit the inflammatory response, a prominent driver of oncogenesis. For instance, upon ischemic injury and brain inflammation, hypothermia has been shown to hinder inflammation by inhibiting microglial and monocyte activation and macrophage infiltration and by decreasing the expression of the intracellular adhesion molecule-1 to block leukocyte recruitment. Furthermore, microglial inhibition upon cold has been shown to protect cells from oxidative stress and damage.^7^^,^^46^^,^^47^^,^^48^^,^^49^ Reduction of pro-inflammatory mediators and modulation of cytokine expression has also been demonstrated upon cold exposure. For instance, patients with encephalopathies due to acute liver injuries showed a significant decrease in pro-inflammatory cytokines (e.g., tumor necrosis factor (TNF) and interleukin (IL)-1β and IL-6) upon exposure to cold.^50^ Importantly, aberrant inflammation, cytokine overexpression, oxidative stress, and DNA damage are important triggers of tumorigenesis, and both boosting nicotinamide adenine dinucleotide (NAD^+^)^51^^,^^52^ and cold exposure have shown similar antitumorigenic and inflammatory properties, suggesting that cold might prevent tumorigenesis and other age-related diseases by similar or converging mechanisms.

Further evidence suggests that crucial factors in the tumorigenic process respond to cold. Among them, cell proliferation and cell cycle progression slow upon cold exposure as a result of impaired polyunsaturated fatty acids, which disturbs the membrane lipid composition.^45^ Additionally, histological characterization of human tumor biopsies exposed to cold showed cytologic changes affecting tumor cells at a comparable rate to those exposed to radiation.^29^^,^^53^

Recently, *in vivo* 4°C cold exposure has been shown to induce brown adipose tissue activation, which competes and deprives cancer cells of glucose, thus inhibiting tumor growth in multiple cancer mouse models such as fibrosarcoma, breast cancer, colorectal cancer, melanoma, pancreatic ductal adenocarcinoma, and secondary liver cancer.^54^^,^^55^

Furthermore, metastasis involves tumor cell detachment, migration, intravasation, and tissue invasion, and its success largely depends on the ability of cells to adhere and establish secondary tumors. *In vitro*, mild hypothermia was found to attenuate breast cancer cell adhesion, preventing intravasation and extravasation, and consequently limiting migration and metastasis.^45^

In 1934, Porfiry Ivanov believed that exposing himself to cold, fasting, and meditation cured his cancer as it “*throws hormone of health into the body, and mobilizes the body defenses*.”^56^ Today, the Win Hof Method (WHM) follows Ivanov’s principle, claiming that the combination of cold exposure, meditation, and breathing techniques (favoring hyperventilation) can strengthen health and prevent diseases by “voluntarily activating the autonomic nervous system to attenuate systemic inflammation.”^57^ Despite limited studies on this method, Kox et al. described that upon experimental endotoxemia, volunteers who employed the WHM showed enhanced epinephrine and anti-inflammatory cytokine IL-10 levels and decreased proinflammatory cytokines (TNF-a, IL-6, and IL-8) when compared to those who were not exposed to the WHM.^57^ Even though cold exposure and meditation may have anti-inflammatory benefits, these studies require further scientific backup, suggesting that extensive research is necessary to provide a strong scientific rationale to back the effectiveness of this method.

For now, these results may be ascribed to the stress response caused by hyperventilation, cold-shock stress (widely known to increase norepinephrine), or to the induction of endotoxemia. Moreover, several studies have suggested health-enhancing benefits of ice-cold water swimming (5°C), which has become a popular endurance sport. Among them, lowering blood pressure, cardiac disease risk prevention, and improved immune response have been suggested, and, similar to the WHM, an increase in cortisol, norepinephrine, catecholamines, and adrenocorticotropic hormones has been reported.^27^ However, the risks and benefits are still controversial, and further studies are required, especially considering that exercise is also a health-enhancing factor.

## Effect of cold in the treatment of acute liver failure (ALF), which predisposes to cancer

Upon ALF, ammonia toxicity is often associated with hepatic encephalopathy, a prevalent neurological disorder and frequent cause of death in cirrhotic patients. ALF also frequently predisposes hepatocellular carcinoma (HCC) development, the most frequent type of liver cancer.^44^^,^^50^^,^^58^ Hypothermia has been shown to extend survival and prevent hepatic encephalopathy in ALF by limiting ammonia entry to the brain, reducing ammonia metabolic enzyme activity, and preventing nitric oxide production, thus lowering cellular oxidative metabolism.^50^^,^^59^ Similar to the ability of hypothermia to improve the detrimental effects of ALF, NAD^+^ precursors have been shown to ameliorate ALF^44^^,^^59^^,^^60^ and liver diseases, including HCC,^52^^,^^61^ suggesting a potential link between NAD^+^ levels and hypothermia, which requires exploration in liver diseases and cancer.

## Effect of cold on lifespan

Not too far from Dr. Hunter’s ideas of offering eternal life upon hypothermia, evidence has described the role of cold in lifespan extension, where a clear correlation between low temperature and longevity has been observed from poikilotherms to homeotherms (Further review in the study by Keil et al.).^62^ Studies performed in *Caenorhabditis elegans* have shown that decreasing the culture temperature increased lifespan,^63^^,^^64^ such that even a 5°C drop in temperature was found to increase lifespan by 75%.^62^^,^^65^ Later, Xiao et al. showed that cold exposure reduced the degree of chemical reactions in *C. elegans*, thus slowing down aging.^66^ Longevity-enhancing effects upon cold exposure were also demonstrated in *Drosophila melanogaster* by regulating mitochondrial metabolism and anti-inflammatory responses,^67^ supporting previous observations that *D. melanogaster* lived twice as long when exposed to 21°C compared to 27°C.^68^

Furthermore, Conti et al. demonstrated that genetically reducing CBT in mice by overexpressing the uncoupling protein 2 in hypocretin neurons (Hcrt-UCP2), contributed to lifespan extension and antiaging properties, similar to those observed upon calorie restriction (CR).^69^^,^^70^ Similarly, Ames dwarf mice (which lack thyroid-stimulating hormone and growth hormone) which present a low CBT have been shown to live approximately one year longer than wild-type normal-size mice.^71^ In cancer, James Arnott believed that using extreme temperatures extended the lifespan of patients with cancer, stating that *“congelation will much prolong it, and prolong it in comfort, by arresting the course of the disease*.*”*^22^ Similarly, *Heterocephalus glaber* or naked mole-rats, considered the longest-lived rodent, are capable of living for more than 30 years; these animals have a low CBT and are exceptionally resistant to cancer,^62^ suggesting a correlation between low CBT, longevity, and cancer prevention.

Notably, CR has been shown to prevent the onset of cancer, diabetes, and cardiovascular and neurodegenerative diseases.^72^ In cancer, CR has been shown to present antitumorigenic effects and reduce cancer morbidity, mortality, and incidence in rodents; inhibit tumoral growth in xenografts; and halve cancer incidence in monkeys. Mechanistically, CR has been shown to boost NAD^+^ and corticosteroid levels, modulate antioxidant systems, reduce oxidative stress markers, inflammation, and inflammatory cytokine expression, and enhance DNA repair, all of which play an essential role in tumor prevention^73^ and have been reported to be modulated following cold exposure. Interestingly, CR has been associated with a reduction in CBT in rodents,^62^^,^^69^ and a decreased CBT has been observed in patients undergoing CR regimens (to 36.64°C ± 0.16) when compared to those exposed to western diets (36.83°C ± 0.2).^62^^,^^74^ Thus, CR protective properties could be due, in part, to the decrease in CBT, which has shown similar properties as previously discussed, suggesting a correlation among low CBT, longevity, and cancer prevention, similar to the observations in naked mole-rats, which display low CBT and cancer resistance.^62^ As aging is a risk factor for cancer, the relationship among hypothermia, CR, and cancer remains to be explored.

## Concluding remarks

It is no coincidence that almost every ancient civilization worldwide, throughout different eras, believed in the healing properties of cold. Based on this historical background arising from superstition and the power of nature, following later primary and methodological experimentation has provided clues for the potential use of cold to treat cancer, wounds, and other diseases. However, the therapeutic potential of cold remains limited and poorly defined. Moreover, despite its promising results in cancer, lifespan extension, cardiac arrest, brain trauma and stroke, neonatal hypoxic enteropathies, spinal cord injury, hepatic enteropathies, inflammation, and ischemic tissue injuries, and despite the variety of experimental animal models, little remains known about how cold exposure and hypothermia favor cancer prevention, progression, and treatment. More importantly, it is unclear why the study and use of hypothermia application in the basic research and clinical treatment of cancer has been somehow forgotten today. Thus, the role of hypothermia and mechanisms of action for cancer treatment warrant further scientific research in the future. Thus, doors must be reopened once again to take advantage of the promising health-enhancing effects of cold, hypothermia, and temperature in disease, and indeed, in a near future to apply cryotherapy alone or in combination with other known therapies (e.g. immune checkpoint blockers) for the treatment of cancers.
